# Paradoxical Cervical Lymphadenitis During Anti-tubercular Therapy in an Immunocompetent Young Female: A Diagnostic Pitfall

**DOI:** 10.7759/cureus.110240

**Published:** 2026-06-04

**Authors:** Ishita Attri, Gunneet Kaur, Rajwinder Dhaliwal, Aakanksha Parik

**Affiliations:** 1 Otolaryngology - Head and Neck Surgery, National Health Mission, Chandigarh, IND; 2 Dental Surgery, Dr. Sharma’s Dental Clinic, Mohali, IND; 3 Medicine, Guru Gobind Singh Medical College and Hospital, Faridkot, IND; 4 Medicine, Ayushman Arogya Mandir, Chandigarh, IND

**Keywords:** anti-tubercular therapy, cervical lymphadenitis, diagnostic challenge, immunocompetent, missed diagnosis, multidrug-resistant tb, paradoxical reaction, tubercular abscess, tuberculosis

## Abstract

A paradoxical reaction (PR) in tuberculosis (TB) is defined as clinical and/or radiological worsening of pre-existing tuberculous lesions or the appearance of new lesions during appropriate anti-tubercular therapy (ATT), despite initial improvement and in the absence of treatment failure, drug resistance, poor adherence, or superinfection. Although more commonly described in HIV-infected or disseminated TB cases, PR can also occur in immunocompetent individuals and may be misinterpreted as disease progression.

We report a case of a 19-year-old immunocompetent female receiving standard first-line ATT for sputum-positive pulmonary TB who developed progressive, painless right cervical lymphadenopathy two months after initiation of therapy. Ultrasonography revealed a large necrotic cervical mass suggestive of a tubercular abscess. Repeated ultrasound-guided fine needle aspiration cytology was inconclusive. Given the absence of systemic symptoms, treatment adherence, and lack of evidence of drug resistance, a diagnosis of PR was considered.

In conclusion, recognition of PR is essential to prevent unnecessary modification of ATT, escalation to second-line therapy, or invasive procedures. Clinicians should maintain a high index of suspicion for PR in compliant patients presenting with new or enlarging lesions during the early months of treatment.

## Introduction

A paradoxical reaction (PR) in tuberculosis (TB) refers to clinical or radiological worsening of existing tuberculous lesions or development of new lesions after initial improvement on effective anti-tubercular therapy (ATT) [1], after alternative causes such as nonadherence, treatment failure, drug resistance, and superinfection have been excluded. The proposed mechanism involves an exaggerated immune response to mycobacterial antigens released during bacterial destruction [2].

PR is most frequently reported in HIV-infected individuals and in disseminated TB. However, it may also occur in immunocompetent patients with pulmonary TB and may be underrecognized in routine clinical practice [3]. Cervical lymphadenitis is among the most common manifestations of PR and can closely mimic treatment failure, multidrug-resistant TB (MDR-TB), or secondary bacterial infection.

PR represents an important diagnostic challenge in routine clinical practice because it can closely mimic treatment failure, MDR-TB, secondary bacterial infection, or even malignancy. Importantly, PR is fundamentally a diagnosis of exclusion, requiring careful evaluation of treatment adherence, microbiological resistance, treatment failure, and superinfection before the diagnosis can be established. Failure to recognize this phenomenon may lead to unnecessary investigations, inappropriate modification of therapy, and increased patient anxiety.

We report a case of paradoxical cervical lymphadenitis in an immunocompetent young female receiving treatment for pulmonary TB, highlighting the diagnostic difficulties, differential considerations, and clinical implications of this uncommon presentation. We also discuss the occurrence of PR in immunocompetent patients despite adequate therapy and review evidence from established peer-reviewed literature to provide additional clinical context.

## Case presentation

**Figure 1:**
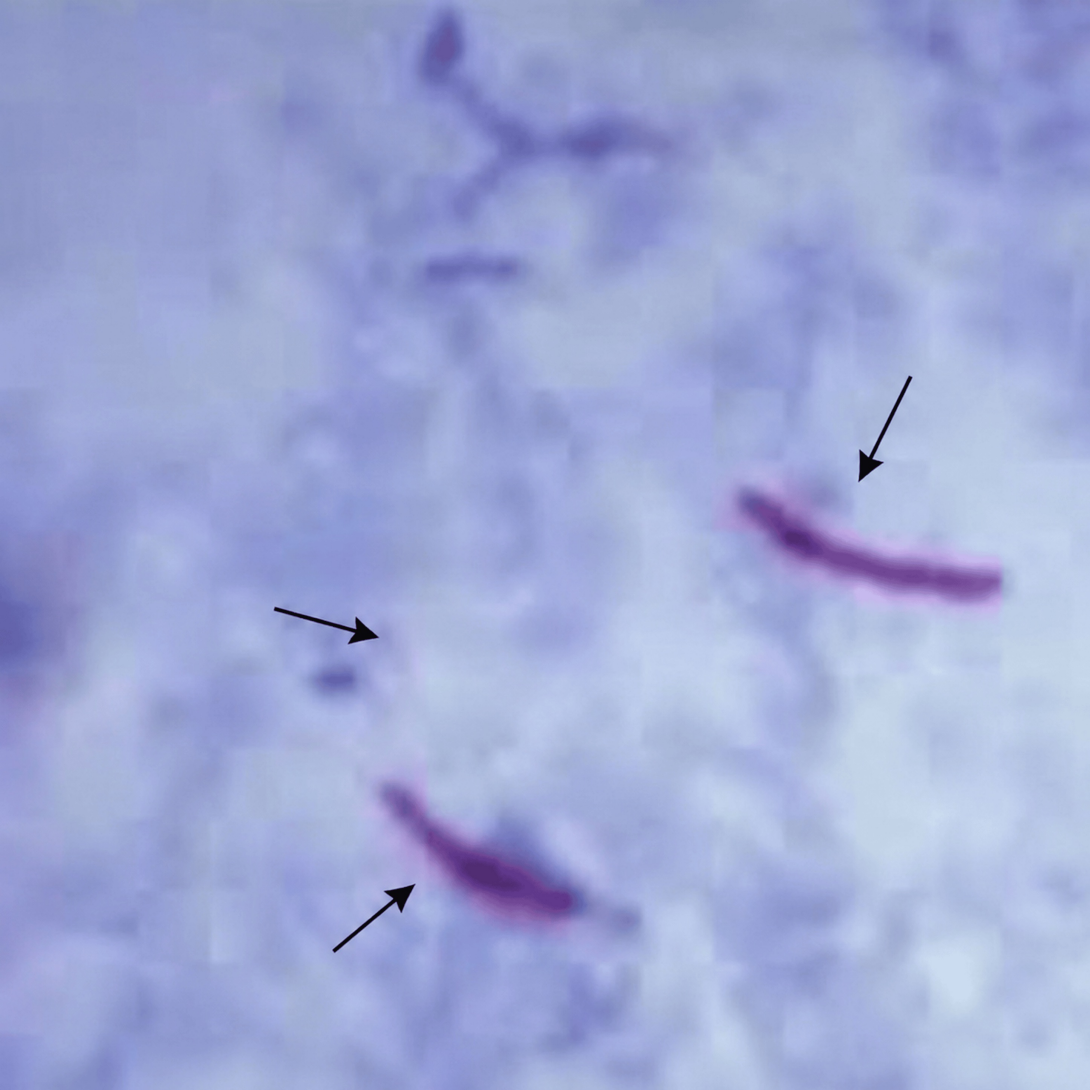
Ziehl-Neelsen staining demonstrated acid-fast bacilli on sputum smear microscopy.

A 19-year-old immunocompetent female from rural Chandigarh, India, was diagnosed with sputum smear-positive pulmonary TB two months before presentation. Sputum microscopy confirmed the presence of acid-fast bacilli consistent with *Mycobacterium tuberculosis*, as shown in Figure 1. Additionally, chest radiography demonstrated bilateral pulmonary infiltrates.

She was initiated on standard first-line ATT consisting of isoniazid, rifampicin, ethambutol, and pyrazinamide. The patient reported strict adherence to therapy and showed initial symptomatic improvement. Adherence to ATT was verified through supervised DOTS (Directly Observed Treatment, Short-Course) treatment at a nearby government dispensary. The patient attended regularly and received medications under direct observation, indicating good compliance. Early symptomatic improvement after initiation of first-line ATT further corroborated adherence to the treatment regimen.

Immunocompetency evaluation was performed. HIV serology and other viral markers were negative. There was no clinical or laboratory evidence of immunodeficiency.

To exclude treatment failure and drug-resistant TB, repeat sputum examinations were performed twice during the period of lymph node enlargement. Repeat sputum smear microscopy, cartridge-based nucleic acid amplification test (CBNAAT/GeneXpert), and mycobacterial culture were all negative. Rifampicin resistance was specifically ruled out by CBNAAT testing. These findings, along with clinical improvement and absence of systemic deterioration, supported continued susceptibility to first-line therapy.

Two months after initiating treatment, she developed a progressively enlarging, painless swelling in the right cervical region. There were no associated systemic symptoms such as fever, cough, loss of appetite, night sweats, or weight loss.

**Figure 2:**
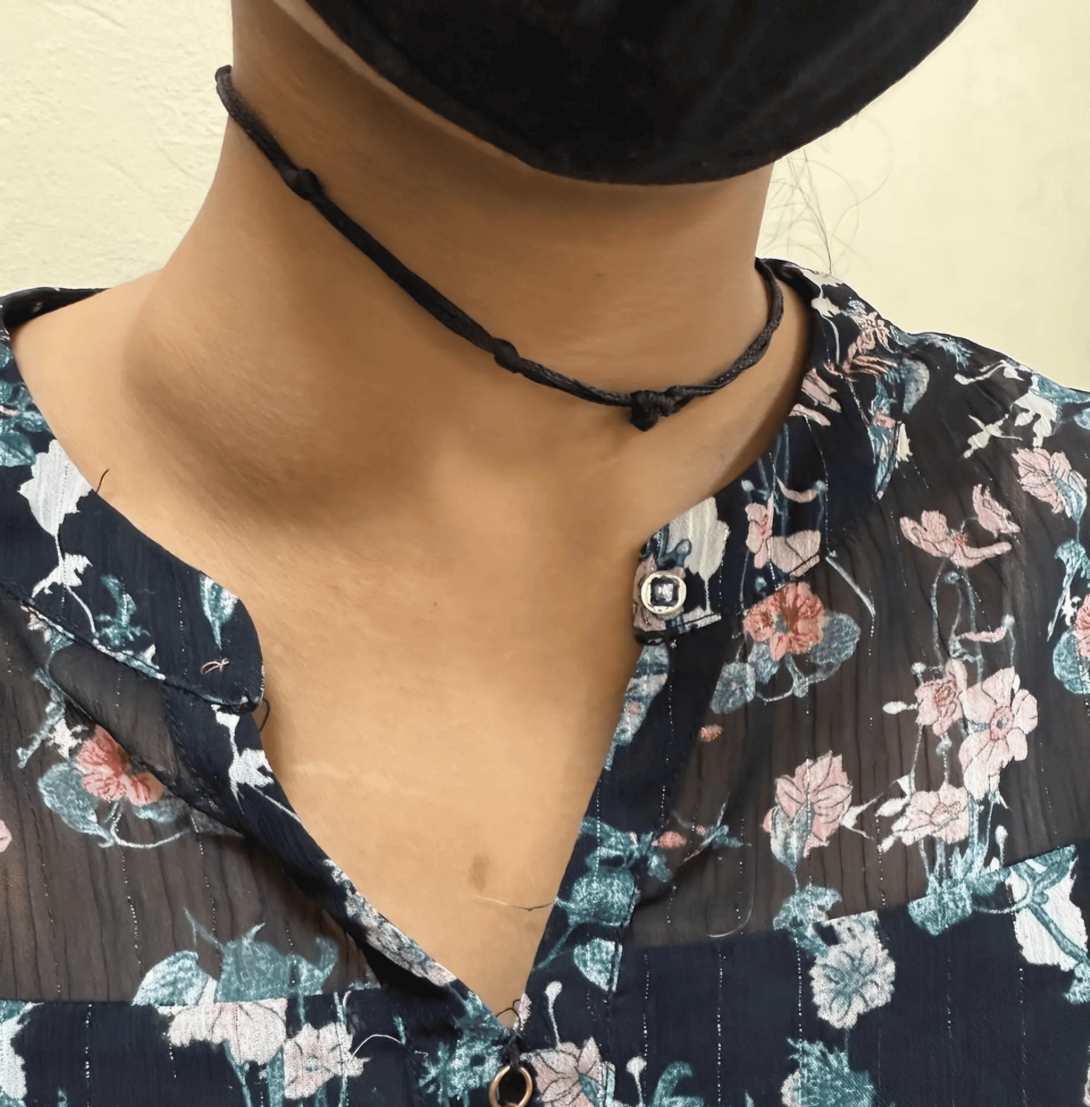
Clinical photograph demonstrating right-sided cervical swelling.

On examination at our center, a single firm, mobile, non-tender lymph node measuring approximately 4.5 × 3 cm was palpated in the right cervical level III-IV region, with no overlying erythema, warmth, fluctuation, or sinus formation, as illustrated in Figure 2. Relevant laboratory investigations are summarized in Table 1. Please note that low hemoglobin is the patient's baseline, as it was found out upon evaluation.

**Table 1:** Lab results.

Parameter	Result	Standard range	Units
Hemoglobin	8.8	12-15	g/dL
Total leukocytes	7500	4000-11000	1/mm^3^
Neutrophils	70	40-80	%
Lymphocytes	20	20-40	%
Monocytes	4	2-10	%
Eosinophils	6	1-6	%
Platelets	3.1	1.5-4.1	lakh/mm^3^

**Figure 3:**
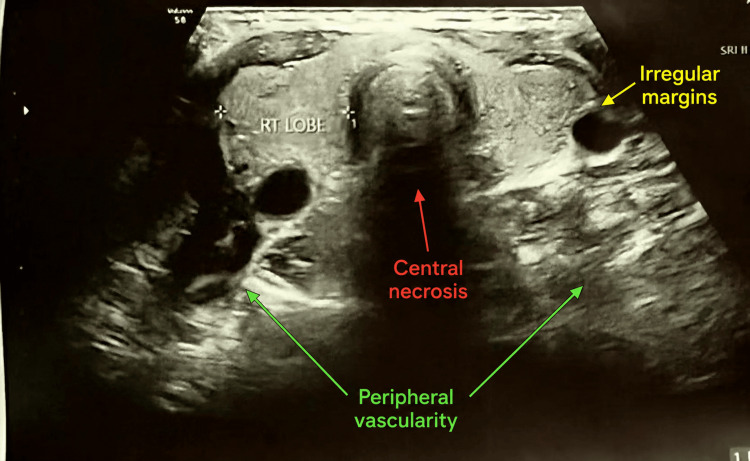
Ultrasound revealed a large, irregular, hypoechoic lesion in the right supraclavicular region.

The patient had undergone numerous investigations at multiple clinics before presenting to our outpatient department. These investigations included neck ultrasonography and ultrasound-guided fine needle aspiration cytology (FNAC). Ultrasonography of the neck demonstrated a large irregular hypoechoic lesion measuring 6.1 × 4.0 × 5.2 cm in the right supraclavicular region (Figure 3), with thick walls, central necrosis, and increased peripheral vascularity, features suggestive of tubercular abscess.

Ultrasound-guided FNAC was repeated twice and revealed predominantly necrotic and inflammatory material, with no evidence of malignancy. The ultrasound findings were also consistent with a PR pattern showing necrotic lymphadenitis with peripheral vascularity.

Follow-up chest radiography did not demonstrate radiological worsening. Serial clinical follow-up demonstrated gradual regression of the cervical lymph node while the patient continued the same ATT regimen, further supporting the diagnosis of PR rather than treatment failure or MDR-TB.

Given the initial clinical improvement, reported adherence, absence of systemic deterioration, and radiological findings consistent with necrotic lymphadenitis, a PR was considered most likely after alternative diagnoses were assessed.

The patient was continued on the same first-line ATT regimen without modification. On subsequent follow-up, a gradual reduction in lymph node size was observed, further supporting the diagnosis of a PR. The patient will be monitored further to observe full recovery.

## Discussion

PR is a recognized but frequently underdiagnosed phenomenon in patients receiving ATT. Brown et al. reported incidence ranges from 6% to 30%, with higher rates among HIV-infected individuals [3]. However, PR also occurs in immunocompetent patients, particularly within the first two to three months of therapy [3,4].

The primary diagnostic challenge lies in differentiating PR from treatment failure, drug-resistant TB, poor adherence, secondary bacterial infection, and malignancy; therefore, the diagnostic workup should be explicitly reported [5,6].

Cervical lymphadenitis developing during therapy is particularly prone to misinterpretation. In such cases, clinicians may unnecessarily escalate to second-line anti-tubercular drugs, prolong or modify treatment regimens, and perform repeated invasive procedures (aspiration, excision, drainage) [5]. This could have happened in our case, too.

The pathophysiology of PR is believed to involve an exaggerated host immune response to residual or released mycobacterial antigens during bacterial killing. This mechanism resembles immune reconstitution inflammatory syndrome (IRIS) observed in HIV-positive patients after initiation of antiretroviral therapy [2,7].

Diagnosis remains clinical and is largely one of exclusion. Key supportive features include initial clinical improvement with ATT, good compliance, no microbiological evidence of resistance, absence of systemic toxicity, and localized inflammatory worsening [5].

Most PRs are self-limiting and require no change in ATT in most cases, depending upon their severity and site [4,8]. Corticosteroids may be considered in severe cases involving the central nervous system or airway compromise.

In our case, the large necrotic cervical lymph node and repeated inconclusive FNACs could easily have led to misdiagnosis as MDR-TB or suppurative abscess requiring drainage. However, awareness of PR and careful clinical correlation prevented unnecessary modification of therapy and further invasive interventions.

## Conclusions

PR should be considered in patients with TB who develop new or worsening clinical, radiological, or pathological lesions during the early months of ATT despite apparent treatment adherence. However, this diagnosis should only be entertained after a thorough evaluation has excluded more common and clinically significant causes, including treatment failure, drug resistance, poor adherence to therapy, superimposed infection, malignancy, and other etiologies of lymphadenopathy.

In the present case, the diagnosis of a PR was supported by the temporal relationship to ATT, clinicoradiological findings, and the systematic exclusion of alternative diagnoses. We acknowledge that the diagnosis was not confirmed histopathologically and was therefore based on clinical and radiological correlation rather than definitive tissue diagnosis. Early recognition of PRs, once alternative causes have been carefully excluded, is essential to prevent misclassification as treatment failure or drug-resistant TB, thereby avoiding unnecessary treatment escalation, invasive procedures, increased healthcare utilization, and patient morbidity. Maintaining a high index of suspicion in appropriate clinical contexts can facilitate timely and appropriate management while minimizing the burden on both patients and healthcare systems.
